# Non-perforated Traumatic Pneumoperitoneum in Maxillofacial Trauma: A Compelling Case Report

**DOI:** 10.7759/cureus.52073

**Published:** 2024-01-10

**Authors:** Divakar Goyal, Aarushi Madaan, Nitin Goyal, Mohd Altaf Mir

**Affiliations:** 1 Trauma Surgery and Critical Care, All India Institute of Medical Sciences, Bathinda, Bathinda, IND; 2 Surgery, Adesh Medical College, Kurukshetra, IND; 3 Radiology, Adesh Medical College, Kurukshetra, IND; 4 Burns and Plastic Surgery, All India Institute of Medical Sciences, Bathinda, Bathinda, IND

**Keywords:** maxillofacial trauma, intraabdominal gas, thoracic trauma, craniofacial trauma, motor vehicle injury

## Abstract

Pneumoperitoneum typically results from intraabdominal gas due to gastrointestinal perforation, with exploratory laparotomy serving as the standard management. While non-surgical causes are well established, instances where pneumoperitoneum lacks an identifiable cause even after laparotomy are sparsely documented. Here, we present a case involving a 22-year-old male who, following a high-velocity road traffic injury resulting in a panfacial fracture, exhibited gross subcutaneous emphysema in the neck, pneumomediastinum, and pneumoperitoneum. This report aims to contribute to the growing understanding of such cases, potentially leading to the development of a management protocol that may help avoid unnecessary laparotomies in similar scenarios.

## Introduction

Pneumoperitoneum is characterized by the presence of intraabdominal gas, primarily attributed to gastrointestinal perforation in most instances. While clinical examinations, such as assessing signs of peritonitis, contribute to the diagnostic process, patients typically undergo emergency exploratory laparotomy following initial resuscitation and radiological evaluations [1-3]. This case report details a young male involved in a high-velocity motor vehicle accident who underwent exploratory laparotomy for pneumoperitoneum. Surprisingly, no evidence of gastrointestinal perforation or diaphragmatic injury was found. This rare case underscores the association of pneumoperitoneum with panfacial fractures and extensive subcutaneous emphysema in the neck region. The teamwork of the radiologist, trauma surgeon, and plastic surgeon saved the life of the young patient.

## Case presentation

**Figure 1:**
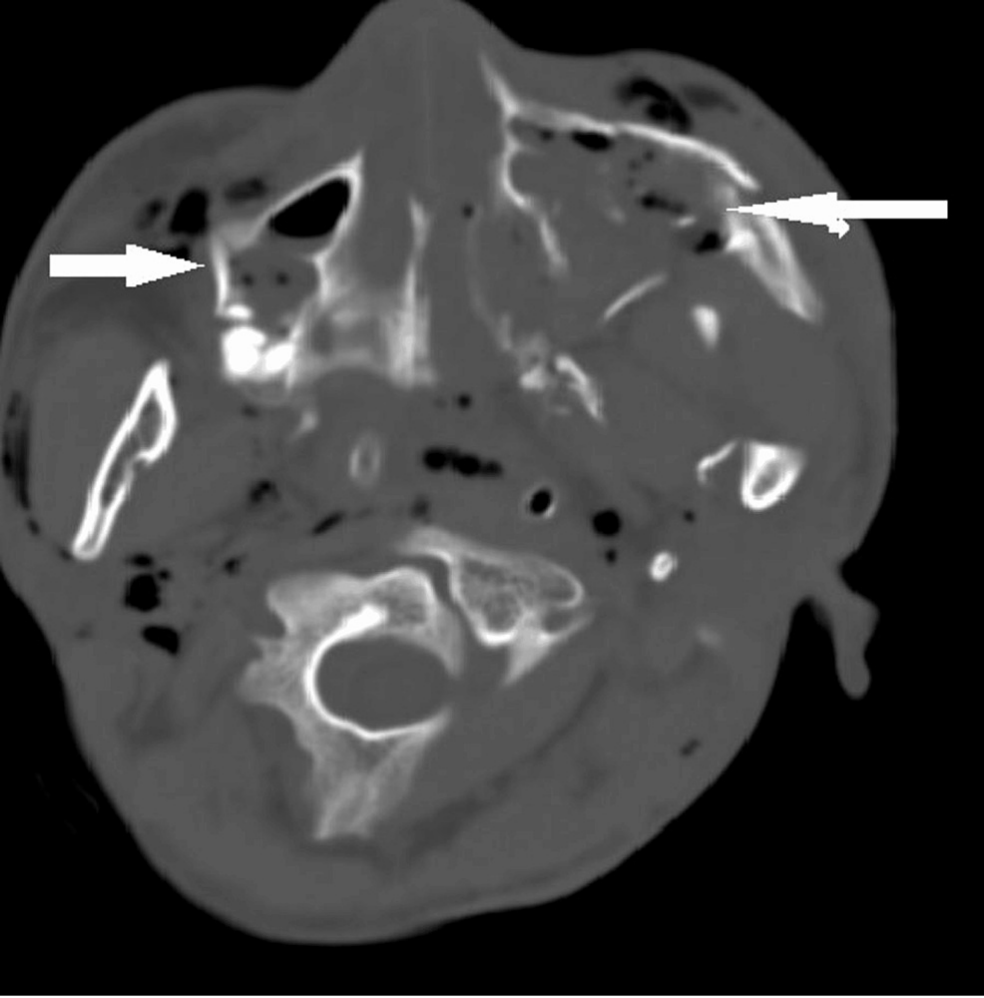
Multiple facial fractures.

**Figure 2:**
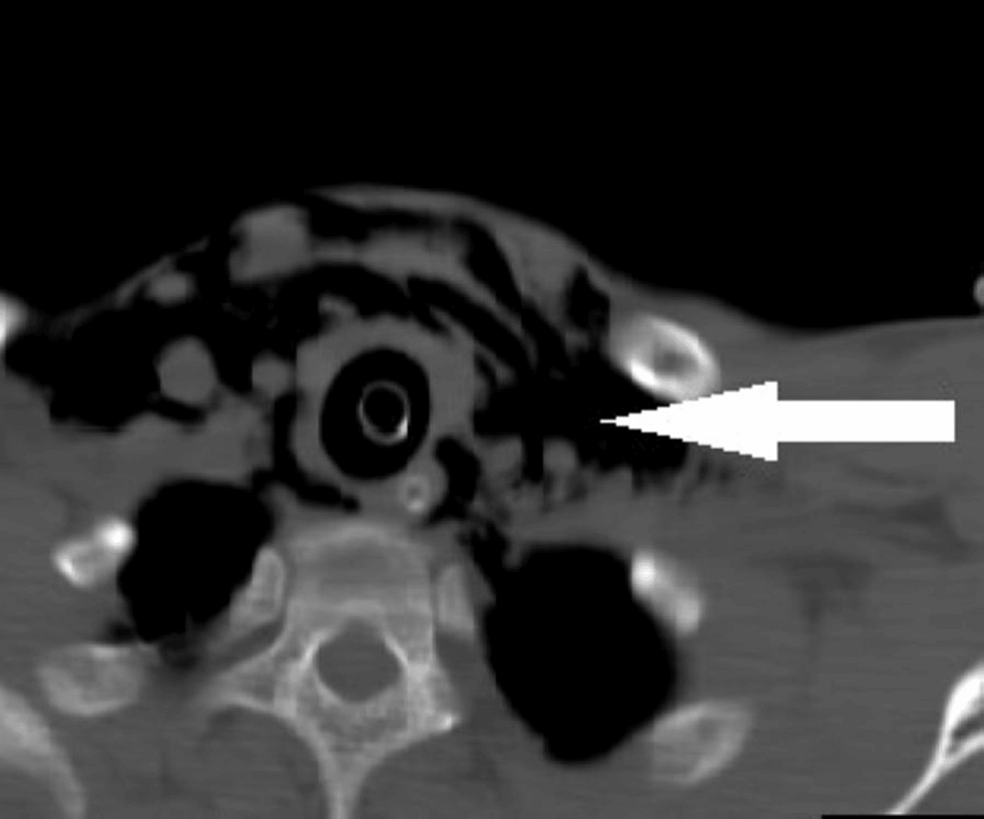
Surgical emphysema in the soft tissues of the neck.

A 22-year-old male was admitted following a high-speed road traffic injury, presenting with panfacial trauma, a fractured right femur, and grade 3 hemorrhagic shock due to blunt trauma to the chest and abdomen. The patient was promptly managed according to Advanced Trauma Life Support (ATLS) principles, receiving resuscitation with intravenous crystalloids and blood products. Given the severity of the shock and the low Glasgow Coma Scale (GCS) score, the airway was secured through oral intubation. Following completion of the primary and secondary surveys, a non-contrast CT scan of the head and cervical spine, including 3D reconstruction, revealed a Le Fort II/III fracture, left temporo-mandibular joint dislocation, and bilateral ramus of the mandible fractures (Figures 1, 2).

**Figure 3:**
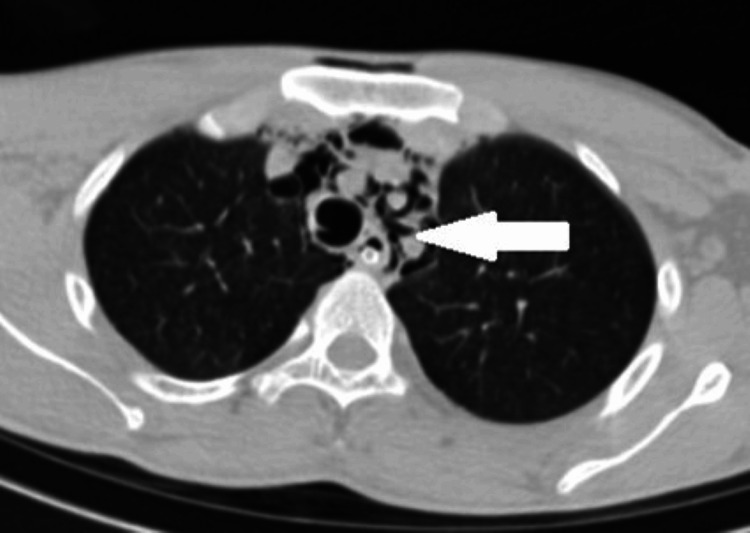
Pneumomediastinum along the branches of the aorta, trachea, and esophagus.

**Figure 4:**
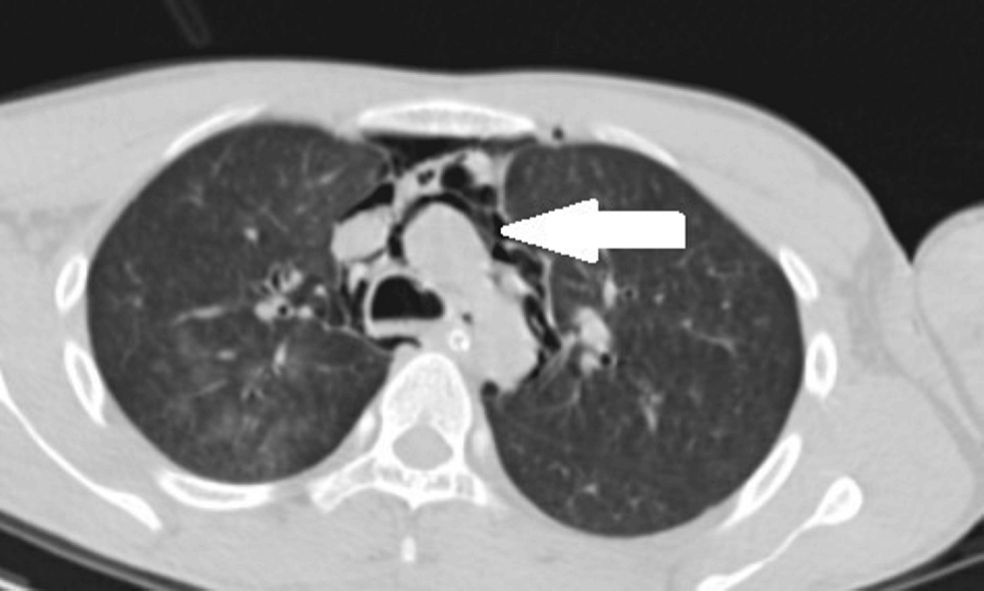
Pneumomediastinum in the superior mediastinum along the mediastinal vessels, trachea, and esophagus.

**Figure 5:**
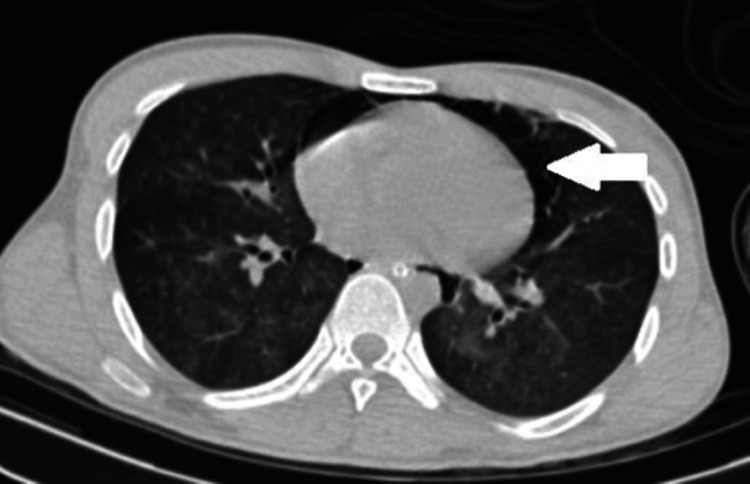
Pneumopericardium.

**Figure 6:**
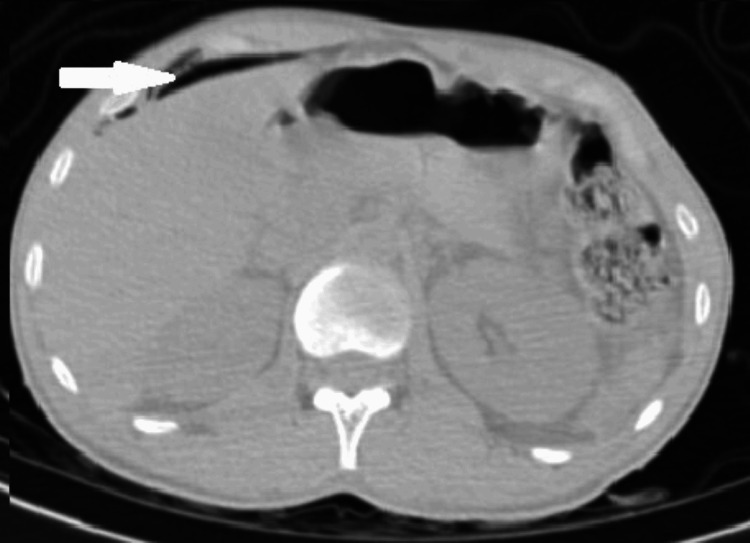
Pneumoperitoneum.

A contrast-enhanced CT scan of the chest and abdomen indicated significant surgical emphysema in the superficial and deep neck spaces, along with pneumomediastinum and pneumopericardium. In addition, air attenuation foci were observed under the right hemidiaphragm, and free intraperitoneal air was noted in the inter bowel space, suggestive of pneumoperitoneum (Figures 3, 4, 5, 6).

The patient received intensive care unit management, including tracheostomy and open reduction with plating for facial fractures by the plastic surgeon. Despite the presence of pneumoperitoneum on the CT scan, exploratory laparotomy by the trauma surgeon revealed no solid organ injury, bowel perforation, or diaphragmatic injury. Serial ECG and 2D echo examinations were conducted to assess for any evidence of blunt cardiac injury. Closed reduction and nailing were performed for the femur fracture. The surgical procedures were done at the same instance through a team approach.

Decannulation occurred on post-injury day 25, and the patient was subsequently discharged. Follow-up over a six-month period showed no evidence of dysrhythmias, with normal 2D echo results, and the patient resumed a regular diet.

## Discussion

Surgical cases of pneumoperitoneum constitute the majority, accounting for 85-90% of occurrences, while non-surgical cases make up 5-15% [1]. Perforated viscus is the most common etiology (85-95%), typically necessitating surgical exploration as the primary treatment. However, non-surgical management has been documented in cases related to mechanical ventilation, amyloidosis, pneumatosis intestinalis, and spontaneous bacterial peritonitis [2-3].

Traumatic pneumoperitoneum cases usually warrant surgical exploration, but studies, such as the one by Currin et al. involving 492 trauma patients, have reported successful non-operative management in two cases using follow-up ultrasound scans. Traumatic perforation commonly involves hollow viscus and diaphragm injuries, with rare instances of intraperitoneal bladder perforation [4,5].

In our case, exploratory laparotomy was performed upon detecting pneumoperitoneum in the contrast-enhanced CT scan, yet no gastrointestinal perforation was found. A case report by Ubukata et al. described idiopathic pneumoperitoneum following high-energy vehicular trauma, emphasizing the absence of obvious gastrointestinal perforation after exploratory laparotomy [6].

The anatomical pathways for air dispersion within the body, particularly through fascial planes, were discussed. Subcutaneous emphysema in the neck can result from air traveling from the mediastinum, while damage to the diaphragm can allow air passage from the chest cavity into the abdomen. The proposed vacuum phenomenon suggests nitrogen gas dissolution due to high-velocity acute fractures or soft-tissue damage, possibly contributing to pneumoperitoneum in our case with pneumopericardium and pneumomediastinum [7,8].

Advances in trauma patient management, particularly the use of whole-body CT scans, have increased the identification of pneumoperitoneum. Studies, such as the retrospective one by Marek et al., highlighted that intra-abdominal free air seen on contemporary CT scans often stems from causes other than severe intra-abdominal damage. When diagnosing pneumoperitoneum, the presence of free fluid, seatbelt sign, or symptoms of bowel damage are crucial indicators of injury and require a thorough examination [9].

Management considerations, as suggested by Ramponi et al., emphasize that clinical evaluation and diagnostic findings determine whether surgical intervention or conservative treatment is appropriate. Monitoring vital signs, leucocytosis, recovery of bowel function, and resolution of discomfort are key factors in assessing patients for conservative therapy [7].

The evolving management of trauma patients may lead to an increased incidence of such cases, necessitating a well-established protocol in the future. While clinical and radiological diagnoses of these entities remain uncommon, trauma and gastro surgeons operating on such patients should be aware of self-limiting pathologies. The potential role of diagnostic laparoscopy in hemodynamically stable patients is encouraged to prevent unnecessary laparotomies. Further research and studies are imperative to establish a definitive protocol for the management of these patients.

## Conclusions

The changing landscape of trauma patient care may result in more cases of similar nature, highlighting the need for a future protocol. Although clinical and radiological diagnoses of these conditions are rare, trauma and gastro surgeons should be mindful of self-limiting pathologies. Encouraging the use of diagnostic laparoscopy in stable patients can help avoid unnecessary laparotomies. Additional research is essential to establish a definitive management protocol for these cases.
